# Three-dimensional analysis of perineural invasion in extrahepatic cholangiocarcinoma using tissue clearing

**DOI:** 10.3389/pore.2023.1611284

**Published:** 2023-06-22

**Authors:** Hirokazu Ogasawara, Tadashi Yoshizawa, Kiyoko Oshima, Kenta Ogasawara, Shunsuke Kubota, Shintaro Goto, Satoko Morohashi, Taiichi Wakiya, Norihisa Kimura, Keinosuke Ishido, Hiroshi Kijima, Kenichi Hakamada

**Affiliations:** ^1^ Department of Pathology and Bioscience, Hirosaki University Graduate School of Medicine, Hirosaki, Aomori, Japan; ^2^ Department of Gastroenterological Surgery, Hirosaki University Graduate School of Medicine, Hirosaki, Aomori, Japan; ^3^ Department of Pathology, Johns Hopkins University School of Medicine, Baltimore, MD, United States

**Keywords:** tissue clearing, three-dimensional, perineural invasion, cholangiocarcinoma, iDISCO

## Abstract

Perineural invasion (PNI) is a characteristic invasion pattern of distal cholangiocarcinoma (DCC). Conventional histopathologic examination is a challenging approach to analyze the spatial relationship between cancer and neural tissue in full-thickness bile duct specimens. Therefore, we used a tissue clearing method to examine PNI in DCC with three-dimensional (3D) structural analysis. The immunolabeling-enabled 3D imaging of solvent-cleared organs method was performed to examine 20 DCC specimens from five patients and 8 non-neoplastic bile duct specimens from two controls. The bile duct epithelium and neural tissue were labeled with CK19 and S100 antibodies, respectively. Two-dimensional hematoxylin/eosin staining revealed only PNI around thick nerve fibers in the deep layer of the bile duct, whereas PNI was not identified in the superficial layer. 3D analysis revealed that the parts of DCC closer to the mucosa exhibited more nerves than the normal bile duct. The nerve fibers were continuously branched and connected with thick nerve fibers in the deep layer of the bile duct. DCC formed a tubular structure invading from the epithelium and extending around thin nerve fibers in the superficial layer. DCC exhibited continuous infiltration around the thick nerve fibers in the deep layer. This is the first study using a tissue clearing method to examine the PNI of DCC, providing new insights into the underlying mechanisms.

## Introduction

Distal cholangiocarcinoma (DCC) is often diagnosed at advanced stage and has poor prognosis [1, 2]. Surgery is a curative treatment option in patients with DCC; however, the prognosis remains poor even after radical resection. Perineural invasion (PNI) is an independent poor prognostic factor after surgery in patients with DCC. Studies examining PNI, a characteristic invasion pattern observed in cholangiocarcinoma as well as in pancreatic cancer [3–5], have revealed that the molecular mechanisms underlying PNI include the activation of nerve growth factor, L1 cell adhesion molecule, matrix metalloproteinases, M3 muscarinic acetylcholine receptor, and hepatocyte growth factor [6–10]. On the other hand, few studies conducted three-dimensional (3D) morphologic analysis of PNI. Maxwell et al. analyzed PNI in extrahepatic cholangiocarcinoma using the 3D construction of serial sections and showed that DCC exhibited continuous branching in the perineural space. However, the resolution of 3D analysis was low, and the observation was limited to a small area around the nerve fibers [11]. Miyashita et al. examined the 3D structure of PNI in Auerbach’s plexus in a cohort of patients with colorectal cancer using the reconstruction of serial sections and reported the presence of two PNI types, PNI with preserved nerve structure and PNI with damaged the nerve structure [12]. However, the structural relationship between nerve fibers and cancer across the full thickness of the bile duct wall using 3D imaging methods has not yet been examined.

In recent years, detailed observation of 3D structures has become possible with the development of tissue clearing methods [13], which have been utilized in pancreatic cancer [14, 15] and extrahepatic cholangiocarcinoma [16] to elucidate invasion mechanisms by 3D observation. In the present study, we aimed to clarify the morphologic characteristics of PNI in DCC using 3D structural analysis with a tissue clearing method.

## Materials and methods

### Patients and tissue samples

A total of 20 cancer tissue specimens, measuring up to 1.5 cm × 1.5 cm × 0.5 cm, in dimensions collected from five patients with DCC, including three and two patients with well-differentiated and moderately differentiated adenocarcinoma, were examined. Additionally, eight non-neoplastic distal bile duct tissue specimens collected from two patients, including one patient with duodenal cancer confined to the mucosa and one patient with duodenal adenoma, were examined as negative controls.

### Fresh tissue preparation

A total of 28 freshly collected bile duct tissue specimens were processed as previously described [14–16]. Briefly, the tissue specimens were fixed in 80% methanol/20% dimethyl sulfoxide (DMSO) overnight. The next day, the specimens were placed in 4% paraformaldehyde and incubated at 4°C for 24 h, followed by dehydration with a series of methanol washes (once each in 50%, 80%, and 90% methanol, and three times in 100% methanol). The specimens were then incubated at 4°C for 60 min, followed by overnight incubation in 66% dichloromethane/33% methanol at room temperature. Next, the specimens were washed twice with 100% methanol and incubated in methanol with 5% hydrogen peroxide at 4°C overnight to oxidize endogenous pigments and autofluorescent proteins. The specimens were rehydrated with a series of washes (90%, 80%, and 50% methanol), washed once with phosphate-buffered saline (PBS), and washed twice, 1 h each, in 1 × PBS/0.2%Triton X-100 (Millipore Sigma, St. Louis, MO, United States). Finally, the specimens were incubated in a permeabilization solution (1 × PBS/20% DMSO/0.2%Triton X-100/0.3 M glycine) at 37°C for 2 days and in a blocking reagent including 1 × PBS/0.2%Triton X-100/10%DMSO/6% donkey serum at 37°C for 2 days.

### Immunolabeling

The specimens were immunolabeled with a mouse monoclonal antibody against S100 (1:250; clone, 4C4.9; cat. no., ab4066; Abcam, Cambridge, United Kingdom) and a rabbit monoclonal antibody against cytokeratin 19 (CK19) (1:200; clone, EP1580Y; cat. no., ab52625; Abcam). For both antibodies, the antibody concentration was gradually increased over 4 days to allow antibody penetration. Additionally, the centrifugal flow was used to promote antibody penetration. The specimens were centrifuged at 300 *g* for 12 h and shaken at 37°C for 12 h, alternating between centrifugation and shaking, during 4 days of antibody incubation. Next, the specimens were washed five times with 1 × PBS/0.2% Tween-20 supplemented with 10 mg/mL heparin, for 60 min each, at room temperature, followed by incubation with Alexa Fluor 488-conjugated AffiniPure F (ab’) 2 fragment donkey antirabbit immunoglobulin G antibody and cyanine 3-conjugated AffiniPure F (ab’) 2 fragment donkey antimouse immunoglobulin G antibody, both from Jackson ImmunoResearch Laboratories (West Grove, PA, United States) for 3 days with protection from light. During immunolabeling with the secondary antibodies, the specimens were centrifuged at 300 *g* for 12 h and shaken at 37°C for 12 h, alternating between centrifugation and shaking. Finally, the specimens were washed five times with 1 ×  PBS/0.2% Tween-20 supplemented with 10 mg/mL heparin, for 60 min each, at room temperature.

### Tissue clearing

The tissues were dehydrated using a series of methanol washes (once each in 50%, 80%, and 90% methanol, and three times in 100% methanol), for 60 min each, followed incubation in 66% dichloromethane/33% methanol once for 3 h and incubation in 100% dichloromethane twice for 15 min. Finally, the tissues were transferred to dibenzyl ether and incubated at 4°C overnight.

### Tissue imaging and analysis

Images of immunolabeled and cleared tissue specimens were acquired with a CellVoyager CQ1 benchtop high-content analysis system (Yokogawa, Tokyo, Japan) using ×4, ×10, and ×20 objectives. The Alexa Fluor 488 signals were visualized using a bandpass filter set with an excitation range of 421/50 nm and an emission range of 480/40 nm to visualize CK19-labeled epithelial cells in normal bile duct and neoplastic cells. The cyanine 3 signals were visualized using a filter set with an excitation range of 550/40 nm and an emission range of 570/50 nm to visualize S100-labeled Schwann cells. Autofluorescence detected with an excitation range of 405/40 nm and an emission range of 460/50 nm was used to visualize the bile duct wall. 3D and surface-rendering image construction was performed using IMARIS software (version 9.4; Bitplane, Zurich, Switzerland).

### Immunohistochemistry

Routinely collected specimens were processed to prepare formalin-fixed/paraffin-embedded (FFPE) tissue blocks, and 4-μm-thick sections were prepared, which were then mounted on saline-coated glass slides for histologic examination. Immunohistochemical examination was performed on deparaffinized sections using the standard avidin-biotin-peroxidase complex method with a BenchMark XT automated immunostainer (Ventana Medical Systems, Tucson, AZ, United States). Neural tissue was identified based on the presence of Schwann cells, which were labeled using a primary rabbit monoclonal antibody against S100 (1:1; clone, EP32; Leica Biosystems, Newcastle, United Kingdom). The sections were washed four times with PBS and incubated with the BOND Polymer Refine Detection reagent (Leica Biosystems) for secondary immunostaining.

### Validation of observations made in cleared tissues

In cases with available fresh tissue, sections next to the fresh tissue harvested for clearing were processed to prepare FFPE blocks, which were sectioned at 4-μm thickness and stained with hematoxylin and eosin (H&E) and S100 for optical microscopic examination. The area of nerve distribution or PNI was marked on H&E-stained sections, and the same lesion in fresh tissue or FFPE tissue was evaluated using 3D images.

This study was performed in accordance with the Declaration of Helsinki for Human Research and was approved by the Ethics Committee of Hirosaki University Graduate School of Medicine (protocol no. 2020-173).

## Results

### Clearing and labeling with antibodies

**FIGURE 1:**
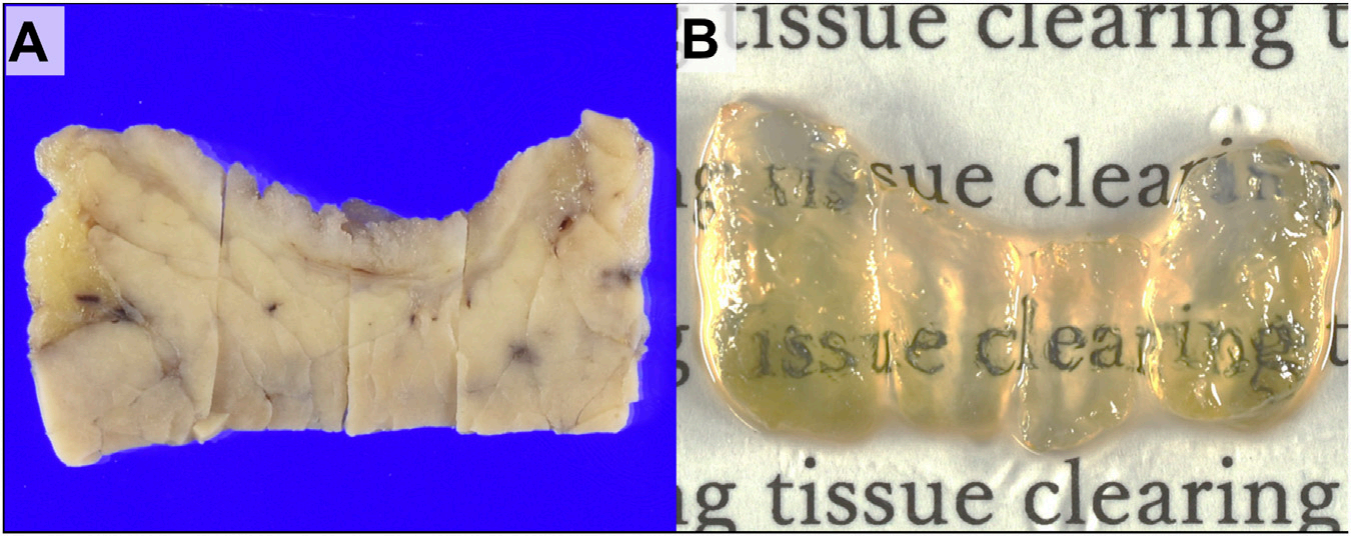
Gross images of the extrahepatic bile duct wall with cholangiocarcinoma. **(A)** Gross appearance of distal cholangiocarcinoma (DCC) before tissue clearing. **(B)** Gross appearance of the specimen after tissue clearing.

All 28 specimens were successfully cleared and immunolabeled to observe the 3D morphology (Figure 1). Fluorescent immunostaining for CK19 allowed us to observe neoplastic and nonneoplastic bile duct epithelium. Neural tissue was also confirmed by observing the presence of S100-labeled Schwann cells coating the periaxonal area. Additionally, the structure of the bile duct wall was observed by autofluorescence. Image acquisition was possible at depths of approximately 200–500 μm.

### 2D and 3D imaging of the normal bile duct wall

**FIGURE 2:**
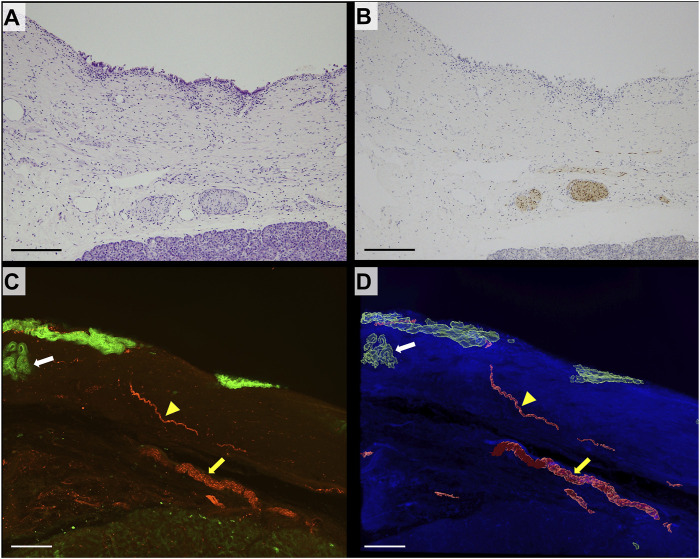
2D and 3D images of nonneoplastic distal bile duct wall. **(A)** 2D image of the hematoxylin/eosin (H&E)-stained section of a non-neoplastic distal bile duct (scale bar, 200 μm). **(B)** 2D image of a section immunohistochemically stained for S100 (scale bar, 200 μm). Thick nerve fibers positive for S100 are seen in the deep layer of the bile duct. **(C)** 3D confocal microscopic images (CK19, green; S100, red; scale bar, 200 μm). **(D)** 3D-rendered image (autofluorescence, blue; scale bar, 200 μm). The large nerve exhibits continuity in the deep layer of the bile duct (yellow arrow). Thin neural fibers extend toward the mucosal surface (yellow arrowhead). Peribiliary glands are identified under the mucosal epithelium (white arrow).

The bile duct in control cases lined with columnar epithelium was observed in 2D H&E-stained sections. Several thick bundles of neural tissue were noted in the deep layer of the bile duct wall adjacent to the pancreas (Figure 2A). S100 immunostaining highlighted the neural tissue in the deep layer of the bile duct; however, detecting it on the superficial layer of the bile duct wall was challenging (Figure 2B). In the 3D images, CK19 fluorescent staining highlighted the biliary epithelium of the bile duct wall. Peribiliary glands, characterized as aggregates of glands, were present beneath the epithelium. The separated bundles of nerve fibers observed in 2D images were continuous in the deep bile duct wall in 3D images. The thin nerve fibers branching from the thick nerve fibers extending toward the mucosal surface was also observed in 3D images (Figures 2C, D).

### DCC in the superficial layer of the bile duct

**FIGURE 3:**
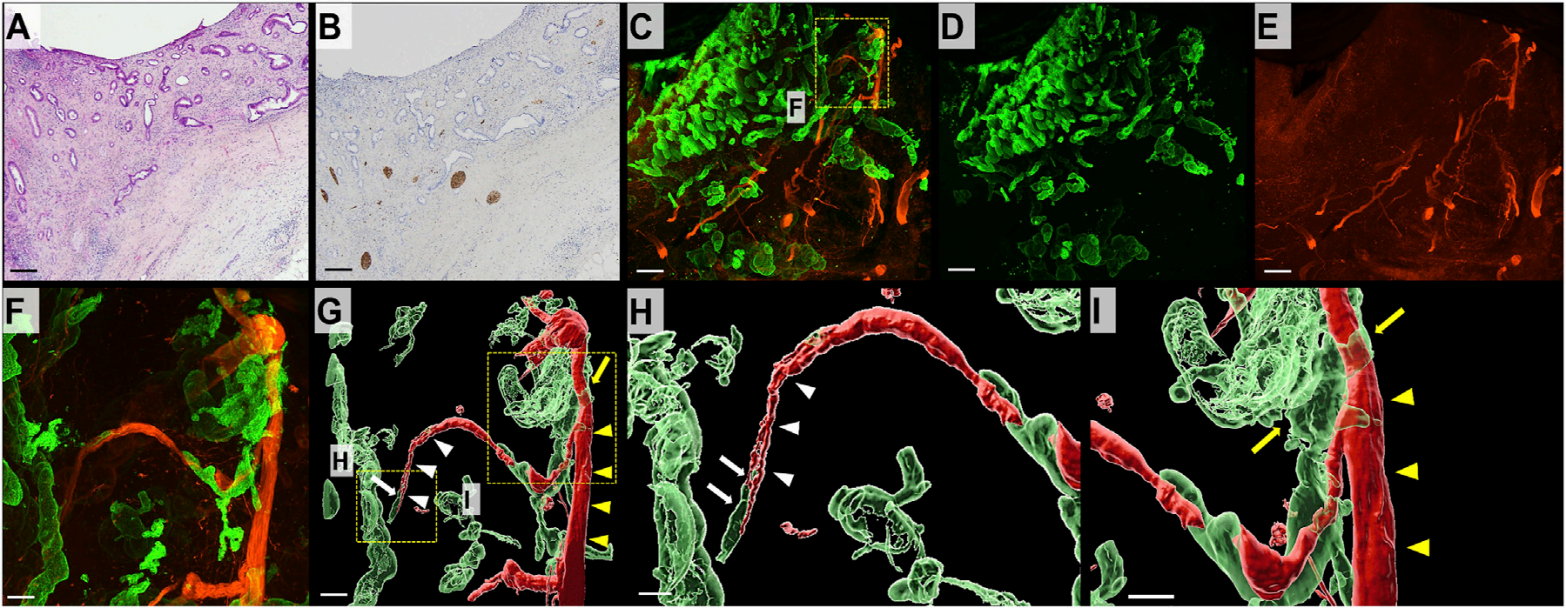
2D and 3D images of the bile duct wall in DCC. **(A)** 2D H&E-stained image of DCC (scale bar, 500 μm). DCC forming tubular structures is infiltrating the bile duct wall. **(B)** 2D image of a section immunohistochemically stained for S100 (scale bar, 500 μm). Thick neural tissue is observed in the deep layer of the bile duct wall. It is difficult to observe neural tissue in the superficial layers. **(C)** 3D microscopic image showing DCC (CK19, green) in combination with neural tissue (S100, red; scale bar, 500 μm). **(D)** DCC forms an interconnecting tubule and invades from the mucosal surface to the deeper layers of the bile duct wall (CK19, green). **(E)** 3D image of neural tissue (S100, red). The bile duct wall with DCC has an extensive network of thin neural tissue. **(F)** Magnified 3D confocal microscopic image (scale bar, 100 μm). **(G)** 3D-rendered image (scale bar, 100 μm). **(H,I)** Magnified 3D-rendered image (scale bar, 150 μm). Thin nerve fibers (white arrowhead) branch from a thick nerve (yellow arrowhead) in the deep mucosal layer. DCC is observed around the thin nerve fiber (white arrow) and exhibits continuous thick nerve fiber (yellow arrow).

In 2D H&E-stained images, DCC forming glands infiltrated the bile duct wall (Figure 3A). Additionally, S100 immunostaining revealed multiple separated bundles of neural tissue within the deep layer of the bile duct; however, superficial nerve tissue was difficult to observe (Figure 3B). In the 3D images, nerve fibers were widely distributed in the superficial layer of the bile duct wall. DCC appeared to form complex interconnecting tubes in the 3D images. The nerve fibers were continuous and branched to form thin nerve fibers widely distributed in the superficial layer of the bile duct wall in 3D images. The thin neural fibers were distributed inside the DCC with a reticular pattern (Figures 3C–E). IMARIS software used to obtain images at higher magnification, and surface rendering was performed to observe the specimens in more detail. As shown in Figures 3F–I, DCC infiltrating along the thin nerve fibers was continuously branching and an area of thick nerve fibers transected by cancer was observed (Supplementary Movie S1).

### DCC in the deep layer of the bile duct

**FIGURE 4:**
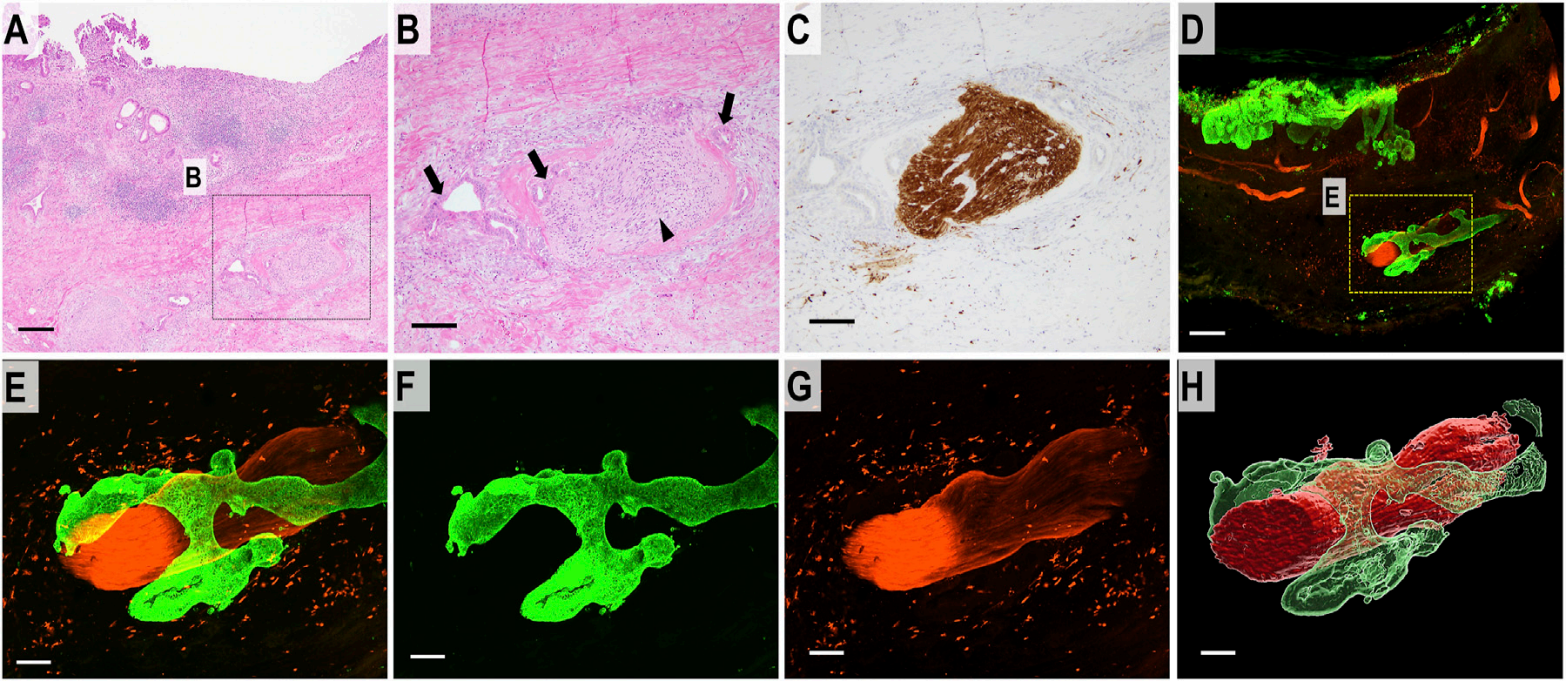
2D and 3D images of DCC with perineural invasion. **(A)** 2D H&E staining image of perineural invasion (scale bar, 500 μm). **(B)** High-magnification 2D image (scale bar, 100 μm). DCC (arrow) is forming glands which infiltrate around the neural tissue (arrowhead). **(C)** 2D image of a section immunohistochemically stained for S100 showing neural tissue in the deep bile duct wall (scale bar, 100 μm). **(D)** Merged 3D confocal microscopic images of perineural invasion. DCC in the full thickness of the bile duct wall (CK19, green). Neural tissue is immunostained with S100 (red) (scale bar, 500 μm). **(E)** Higher-magnification merged 3D confocal microscopic images showing perineural invasion. **(F)** 3D confocal microscopic image of DCC (CK19, green). **(G)** 3D confocal microscopic image of neural tissue (S100, red). **(H)** 3D-rendered image of DCC continuously invading the surrounding perineurium (scale bar, 150 μm).

The 2D H&E-stained images showed separate large bundles of nerve fibers in the deep layer of the bile duct. DCC forming glands were present adjacent to the nerve fibers and confirmed the diagnosis of PNI. S100 immunostaining showed thick bundles of neural tissue (Figures 4A–C). The 3D images revealed that the separate bundles of neural tissue observed in the 2D images were continuous in the deep layer of the bile duct wall. DCC surrounded and extended along the neural tissue in the bile duct wall (Figures 4D–H) (Supplementary Movie S2).

## Discussion

This was possibly the first study to examine the 3D structural relationship of DCC and nerve fibers in full-thickness bile duct specimens, and the results have revealed several findings. First, although the 2D analysis of H&E-stained sections showed PNI limited to the thick nerve fibers in the deep layer, the 3D analysis of the tissue specimens processed with tissue clearing revealed that DCC had more complex nerve fibers on the surface of the bile duct than in the normal bile duct wall and that these structures continuously branched from thick nerve fibers in the deep layer of the bile duct. Second, DCC invaded the bile duct wall by forming an interconnecting tube from the epithelium and extended around the thin nerve fibers in the superficial layer. Finally, DCC exhibited a contiguously invasive pattern by surrounding the thick nerve fibers in the deep layer of the bile duct.

A previous 2D study reported that the nerve fibers are abundantly distributed within the deep layer of the distal bile duct but that only a small amount of nerve fibers were identified within the superficial layer [17]. PNI in the superficial bile duct wall was not considered a frequent finding, and that it was reported to occur mainly in the deep layer [17]. However, our 3D analysis has revealed that more nerve fibers are distributed in the superficial layer of the bile duct in DCC samples compared with the non-neoplastic bile duct wall; PNI was observed even in the superficial layer. Through 2D and 3D reconstruction of prostatectomy specimens, Ayala et al. reported that mouse dorsal root ganglia extended neural tissue toward prostate cancer and formed prostate cancer-associated axons with nerve enlargement or increased nerve density [18, 19]. These studies indicated that the mechanism of PNI involved active and reciprocal interactions between cancer cells and adjacent nerves/ganglions during cancer progression. Although the small number of cases in the present study did not allow statistical analysis to compare the distribution of nerve fibers in the bile duct wall between the normal and DCC cases, our findings are consistent with their experimental results.

Previous studies using 3D imaging analysis of extrahepatic cholangiocarcinoma using reconstruction of serial sections demonstrated that cancer cells extended in a perineural space continuum [11, 19]. However, the area visible by the 3D construction of the serial sections in these studies was small and could not fully reveal the architecture of the entire thickness of the bile duct. Our 3D analysis using specimens processed with tissue clearing enabled the analysis of the full thickness of the bile duct wall from the surface to the deep layer and clarified that the thin nerve fibers was widely distributed on the mucosal side and that cancer cells infiltrated this thin nerve fibers. Additionally, the cancer cells continuously wrapped around the nerve fibers and spread widely into the bile duct wall alongside the thick nerve fibers in the deep layer. Our 3D observations raise the possibility that DCC, while invisible in H&E-stained sections, might remain around the thin nerve fibers at the surface of the bile duct wall in cases where PNI is present near the margin of bile duct resection, explaining the poor prognosis associated with PNI in patients with DCC.

The pathways of abdominal spread of gastrointestinal cancer can be direct, perineural, lymphatic, hematogenous, or peritoneal. Whether the perineural space is an independent compartment or is connected to a lymphatic channel has not yet been confirmed. A 3D study using the reconstruction of serial sections demonstrated the relationship between the nerve fibers and adjacent lymphatics and suggested that DCC in the perineural space was a result of direct invasion from the lymphatics. The authors reported tumor cell clusters floating within the dilated lymphatic vessel adjacent to the nerve fibers and hypothesized that these cells broke into the perineural space [20]. However, the resolution of the reconstructed 3D images of the serial tissue sections was not sufficient to visualize the tumor cell clusters breaking into this perineural space. The present study did not analyze the structural relationship between PNI and lymphatics; however, the 3D images showed the continuous infiltration of DCC in the perineural area, suggesting that PNI was an independent route distinct from invasion through lymphatic vessels or vessels or direct invasion and that PNI provided a favorable environment for DCC spread Future studies should include the comprehensive analysis of the relationship between DCC and neural, lymphatic, and vascular structures using 3D images of specimen after tissue clearing, which may provide new insights into the mechanism of PNI.

## Data Availability

The raw data supporting the conclusion of this article will be made available by the authors, without undue reservation.

## Supplementary material

SUPPLEMENTARY MOVIE S1 Perineural invasion of the images in the superficial layer of the bile duct.

SUPPLEMENTARY MOVIE S2 Perineural invasion of the images in the deep layer of the bile duct.
